# Plasma‐Engineered Polyethylene‐Reinforced Anion Exchange Membranes With Low Ionic Resistance and Low Hydrogen Crossover for Water Electrolysis

**DOI:** 10.1002/advs.77467

**Published:** 2026-08-29

**Authors:** Sungjun Kim, Yeram Shin, Minseop So, Jungwon An, Dong‐Chan Kim, Jang Yong Lee, Segeun Jang

**Affiliations:** ^1^ Hydrogen Energy Research Center Korea Research Institute of Chemical Technology (KRICT) Daejeon Republic of Korea; ^2^ School of Mechanical Engineering Kookmin University Seoul Republic of Korea; ^3^ Department of Chemical Engineering Konkuk University Seoul Republic of Korea

**Keywords:** anion exchange membrane water electrolysis (AEMWE), impregnation, plasma treatment, porous polyethylene (PE), reinforced composite membrane (RCM)

## Abstract

Anion exchange membrane water electrolysis (AEMWE) enables low‐cost green hydrogen production but remains limited by the delicate balance among ionic resistance, gas crossover, and mechanical durability in hydrocarbon‐based membranes. Although reinforced composite membranes (RCMs) alleviate this limitation, conventional expanded polytetrafluoroethylene (ePTFE) reinforcements suffer from fluorocarbon–hydrocarbon interfacial incompatibility, resulting in incomplete ionomer impregnation. We report a plasma‐modified polyethylene (PE) reinforcement that promotes hydrocarbon ionomer infiltration within dense submicron pores. Oxygen plasma treatment introduces polar functional groups on the PE surface, enhancing wettability and capillary‐driven impregnation without altering morphology. The plasma‐treated PE‐based RCM achieves high strength, low swelling, and reduced hydrogen crossover, exhibiting a crossover current density of 0.133 mA cm^−2^ at ∼30 µm thickness, over fourfold lower than a non‐reinforced membrane. Under practical operation, the anode hydrogen concentration remains well below the explosion safety limit, while the AEMWE performance is comparable to an optimized alcohol‐mediated PTFE‐based RCM, despite its denser pore structure. A membrane selectivity factor coupling ohmic resistance and hydrogen crossover quantifies this balance. The plasma‐treated PE‐based RCM exhibits the highest membrane selectivity factor, 3.26‐ and 2.26‐fold higher than the non‐reinforced and ePTFE‐reinforced membranes, respectively. Continuous operation for 1000 h at 1.0 A cm^−2^ confirms long‐term durability.

## Graphical abstract

Oxygen plasma functionalizes porous polyethylene, enabling complete hydrocarbon ionomer infiltration within a dense submicron pore network. The resulting reinforced composite membrane simultaneously enhances mechanical robustness and suppresses hydrogen permeation at ultrathin thickness. An integrated performance index confirms the effectiveness of the plasma‐engineered architecture in achieving both high ionic conductivity and low gas permeability.

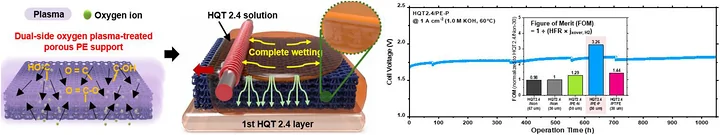

## 1 Introduction

The rapid expansion of renewable energy and global carbon neutrality initiatives has intensified the demand for green hydrogen as a clean and dispatchable energy carrier [1, 2]. Among various hydrogen production technologies, anion exchange membrane water electrolysis (AEMWE) has emerged as a promising alternative to proton exchange membrane water electrolysis because alkaline operation enables the use of earth‐abundant catalysts and non‐precious hardware, offering potential cost reduction while maintaining high current‐density operation [3, 4, 5, 6, 7, 8]. Despite these advantages, the practical deployment of AEMWE remains constrained by membrane‐related limitations that directly govern efficiency, safety, and long‐term stability [9, 10, 11, 12]. For reliable operation under dynamic conditions associated with renewable energy inputs, anion exchange membranes (AEMs) must simultaneously exhibit high ionic conductivity, low gas permeation, and robust mechanical stability [13]. However, these properties are intrinsically coupled by strong trade‐offs. In particular, the lower mobility of hydroxide ions compared with protons necessitates high ion‐exchange capacities, which often induce excessive swelling, dimensional instability, and increased hydrogen crossover [5, 9]. These competing requirements make it difficult to design membranes that simultaneously satisfy performance, durability, and safety criteria.

Reinforced composite membranes (RCMs) have therefore been widely adopted to mitigate these trade‐offs by incorporating a porous support into the ion‐conducting matrix [14, 15]. Reinforcement enables membrane thinning while enhancing mechanical robustness and gas‐barrier properties, thereby reducing ohmic resistance and improving overall electrolyzer performance. In PEM systems, expanded polytetrafluoroethylene (ePTFE) reinforcements have been successfully implemented owing to their chemical stability and mechanical strength [16]. However, this strategy is not directly transferable to AEMWE, because hydrocarbon‐based anion‐conducting polymers, particularly aryl‐ether‐free structures required for alkaline stability, exhibit poor interfacial compatibility with fluorocarbon‐based ePTFE substrates [11, 17, 18, 19, 20]. This incompatibility hinders uniform ionomer impregnation, limiting the formation of thin, dense, and defect‐free RCMs. Although wet‐chemical surface treatments such as alcohol‐assisted impregnation or catechol‐based coatings have been proposed [21, 22, 23, 24], these approaches introduce process complexity and limited scalability for industrial manufacturing. Porous polyethylene (PE) has recently attracted attention as an alternative reinforcement material due to its low cost, excellent mechanical properties, chemical simplicity, and established large‐scale manufacturing infrastructure [25, 26]. However, porous PE typically exhibits lower porosity and smaller pore sizes than ePTFE, which restrict ionomer infiltration and complicate the fabrication of high‐performance RCMs [27, 28]. This dense pore architecture, while often regarded as a structural disadvantage for ionomer impregnation, can become a functional advantage if complete and uniform infiltration is achieved, offering enhanced mechanical robustness and gas‐barrier capability at reduced membrane thickness. Overcoming this limitation requires effective interfacial engineering to enable complete ionomer impregnation within the dense pore network of PE.

In this study, we address this challenge by developing a plasma‐modified porous PE reinforcement that enables efficient infiltration of a high‐performance aryl‐ether‐free hydrocarbon ionomer (HQT2.4) [29]. Oxygen plasma treatment introduces oxygen‐containing functional groups onto the PE surface without altering its bulk morphology, dramatically enhancing wettability and thereby enabling complete ionomer impregnation within the dense submicron pore network of PE, which is otherwise inaccessible using conventional hydrocarbon‐based processing routes. This strategy enables the fabrication of ultrathin (∼30 µm), fully impregnated PE‐based RCMs with superior mechanical stability, suppressed swelling, and exceptionally low hydrogen crossover. Furthermore, a membrane selectivity factor is introduced as a condition‐specific comparative descriptor to quantitatively evaluate the coupled balance between ionic resistance and hydrogen crossover, demonstrating that the plasma‐modified PE architecture substantially improves this balance and thereby mitigates a long‐standing limitation of AEMWE membranes.

## 2 Results and Discussion

### 2.1 Characterization of Porous Substrates and Plasma Modification Effect

**FIGURE 1:**
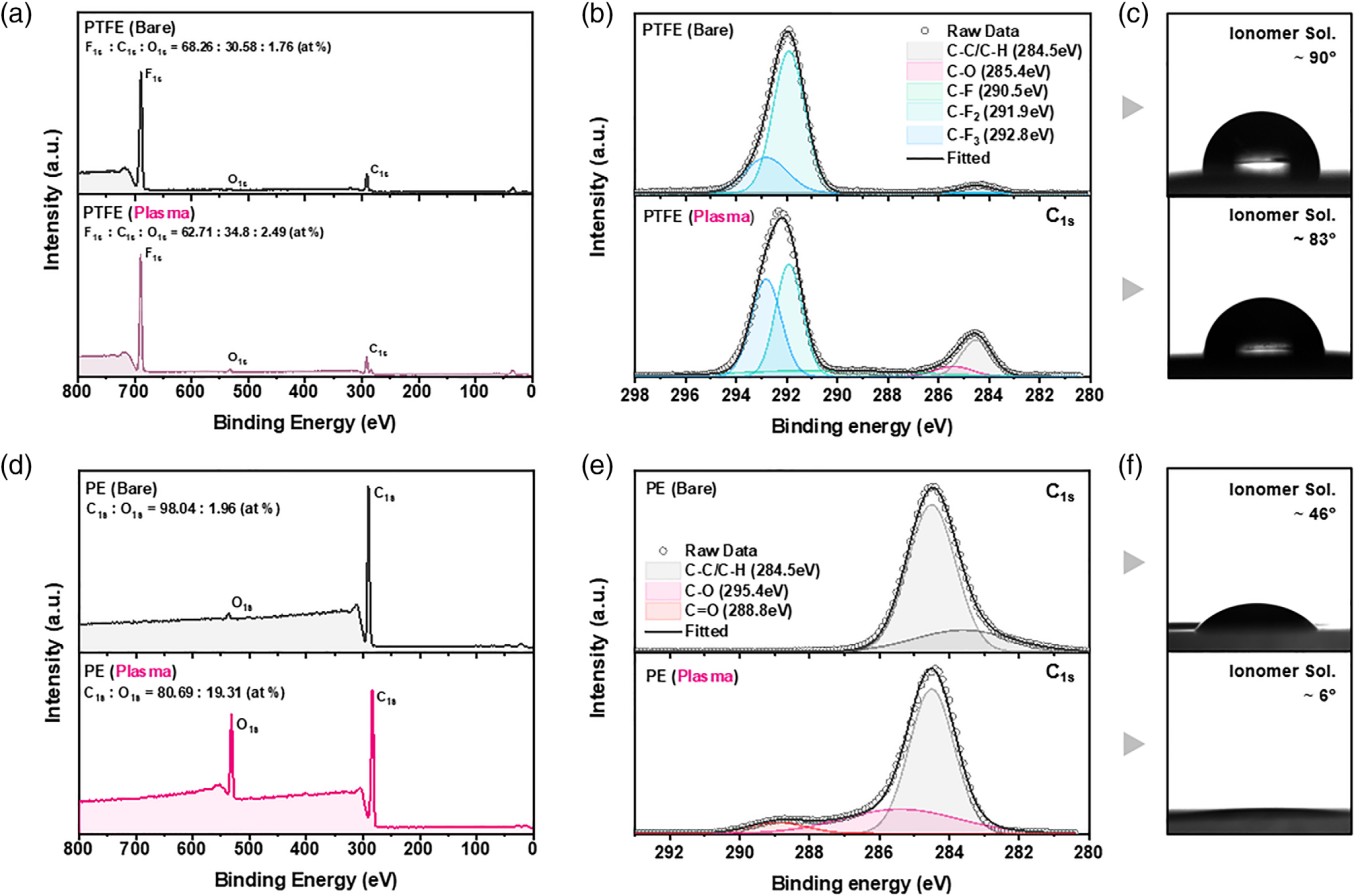
(a, d) Wide‐scan XPS spectra, (b, e) high‐resolution C_1s_ spectra of bare and plasma‐treated porous PTFE and PE substrates. (c, f) Contact angles of HQT2.4 ionomer solution on bare and plasma‐treated porous PTFE and PE substrates.

Reinforcement using ePTFE has been widely adopted in perfluorosulfonic acid ‐based membranes; however, its applicability to hydrocarbon‐based AEMs is fundamentally limited by poor interfacial compatibility and the chemical inertness of fluorocarbon surfaces. Figures S1a,b show that pristine ePTFE exhibits high contact angles toward both water and the HQT2.4 ionomer solution (130° and 90°, respectively), reflecting its low surface energy and non‐wetting nature. After oxygen plasma treatment, the contact angles decreased only marginally (119° for water and 83° for ionomer solution), indicating limited surface activation. This behavior was confirmed by X‐ray photoelectron spectroscopy (XPS) (Figure 1a), where the oxygen content increased only slightly from ∼1.76 to ∼2.49 at.%. High‐resolution C_1s_ spectra (Figure 1b) and O_1s_ spectra (Figure S2a,b) further indicate negligible incorporation of polar functionalities on the ePTFE surface.

In contrast, porous PE substrates exhibited substantially lower intrinsic contact angles (86° for water and 46° for ionomer solution; Figure S1c,d), consistent with favorable hydrocarbon–hydrocarbon affinity between PE and HQT2.4. Oxygen plasma treatment dramatically enhanced PE surface wettability, reducing contact angles to 24° for water and ∼6° for ionomer solution. XPS survey spectra (Figure 1d) reveal a pronounced increase in oxygen content from ∼1.96 to ∼19.31 at.% after plasma treatment, accompanied by the emergence of C─O and C═O components in the high‐resolution C_1s_ spectra (Figure 1e) and corresponding changes in the O_1s_ spectra (Figure S2c,d). This strong plasma response is attributed to the oxidizable ─CH_2_─ backbone of PE, which enables efficient incorporation of oxygen‐containing functional groups. Importantly, the plasma‐treatment configuration used in this study enabled effective surface oxidation of PE while preserving the porous framework [30]. To verify this, we compared the plasma‐treatment condition with a reactive ion etching (RIE) condition using the same capacitively coupled plasma (CCP) system. As illustrated in Figure S3, plasma mode places the substrate on the grounded electrode, allowing reactive oxygen species to reach the porous PE surface primarily through diffusion while minimizing direct ion bombardment and physical etching. In contrast, RIE mode places the substrate on the powered electrode, where a self‐bias voltage accelerates ions toward the surface and promotes significant physical etching. Detailed operating principles and processing parameters for both modes are provided in Figure S3, Table S1, and the Experimental Section. As shown in Figure S4, the plasma treatment condition used in this study (O_2_ plasma, 2 min) resulted in negligible thickness variation and preserved the porous morphology of the PE support. In contrast, RIE‐mode treatment caused a significant thickness reduction from 9.22 to 6.68 µm after 2 min of treatment, accompanied by severe pore collapse and structural deterioration. These results further confirm that the plasma treatment employed in this work effectively introduces oxygen‐containing functional groups while maintaining the structural integrity of the porous PE framework.

**FIGURE 2:**
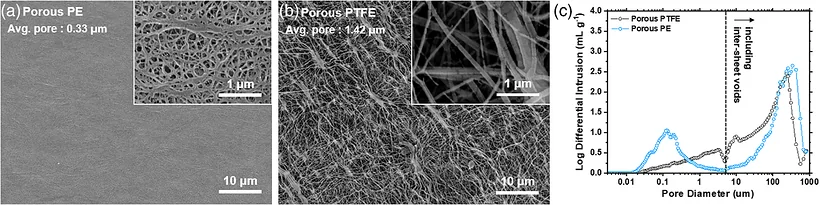
Surface SEM images of porous (a) PE and (b) PTFE substrates. (c) Pore size distribution of porous PE and PTFE substrates obtained from mercury intrusion porosimetry.

While porous PE offers clear advantages in interfacial compatibility, its pore architecture presents a structural challenge for ionomer impregnation. As shown in Figure 2 and Figure S5, porous PE exhibits a dense morphology with tightly packed submicron pores (average diameter ∼0.33 µm), whereas ePTFE features a highly open fibril–node network with larger interconnected pores (average ∼1.42 µm). Mercury intrusion porosimetry (MIP) profiles (Figure 2c) show a dominant submicron intrusion peak for PE and a broad micron‐scale distribution for PTFE. The MIP profiles are consistent with scanning electron microscopy (SEM) observations and clearly highlight the substantially more compact pore network of PE. From a structural standpoint, the dense PE pore network is intrinsically less favorable for ionomer penetration than ePTFE; however, it offers advantages in mechanical stability and gas‐barrier properties. Plasma‐induced surface activation is therefore essential for enabling effective ionomer impregnation into the constrained PE pore network, transforming this structural limitation into a functional advantage for fabricating thin and robust reinforced composite membranes.

### 2.2 Fabrication of RCMs and Their Properties

**FIGURE 3:**
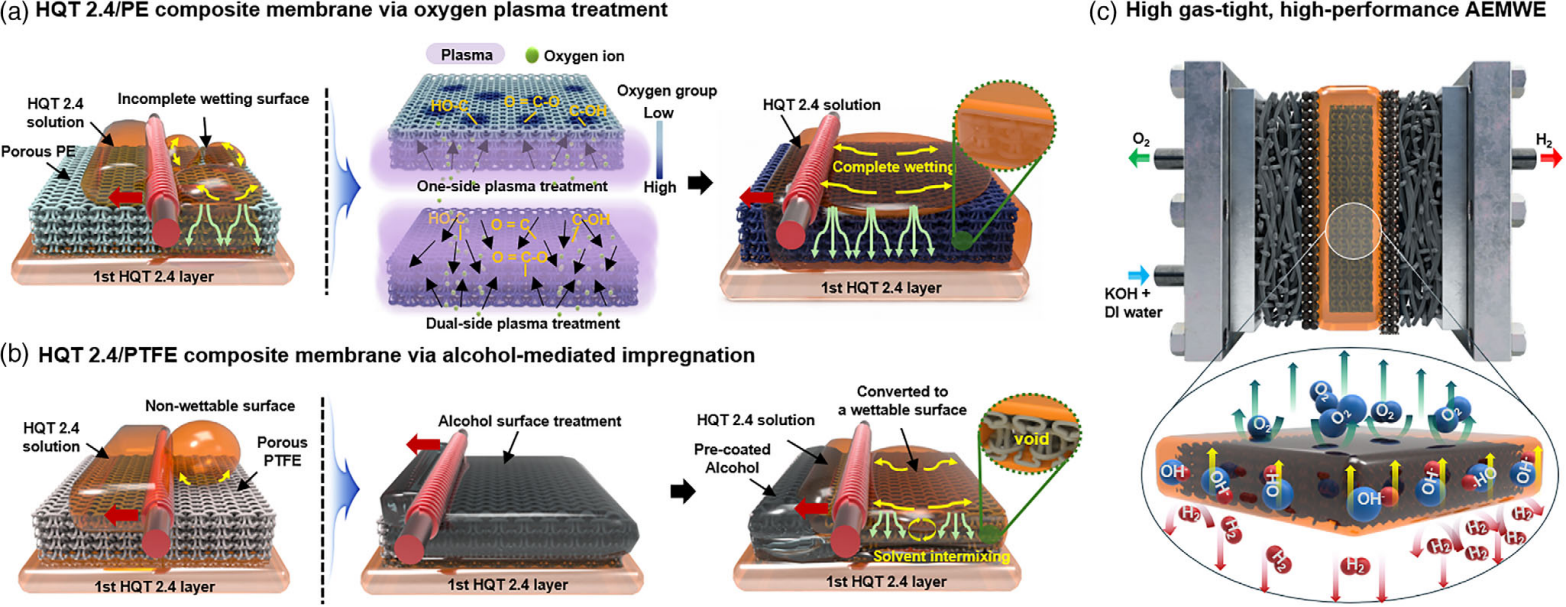
Schematic illustration of the fabrication process for (a) porous PE‐based RCMs and (b) PTFE‐based RCMs using HQT2.4 ionomer. (c) AEMWE with porous PE‐based RCMs.

Figure 3 schematically illustrates the fabrication procedures of RCMs using porous PE and PTFE substrates. In both cases, fabrication was initiated by casting a first HQT2.4 ionomer layer onto a glass substrate. For PE‐based RCMs, the porous PE support was either used in its pristine state or subjected to oxygen plasma treatment prior to coating (Figure 3a). Pristine PE exhibits partial wetting toward the hydrocarbon ionomer, resulting in island‐like spreading and non‐uniform impregnation through the pore network. After plasma modification, oxygen‐containing functional groups are introduced on the PE surface, transforming it from partially wettable to fully wettable and enabling uniform, continuous, and capillary‐driven infiltration throughout the dense PE pore network. For one‐side plasma treatment, reactive oxygen species penetrate through the porous structure and induce surface functionalization along the thickness direction. However, as schematically illustrated in Figure 3a, plasma‐induced modification is expected to decrease with increasing depth from the plasma‐exposed surface, resulting in a gradient of oxygen‐containing functional groups across the porous PE support. Therefore, dual‐side plasma treatment was employed to achieve more uniform functionalization throughout the entire porous PE support prior to ionomer impregnation, thereby facilitating more uniform and continuous ionomer infiltration through the porous network. In contrast, direct impregnation into PTFE is fundamentally infeasible due to its extremely poor wettability toward hydrocarbon‐based ionomers, as schematically illustrated in Figure 3b. To enable comparison, an alcohol‐mediated impregnation strategy was employed for PTFE‐based RCMs [22]. Temporary ethanol wetting allows limited ionomer infiltration; however, evaporation restores the intrinsic fluorocarbon–hydrocarbon incompatibility, leading to interfacial separation and discontinuous ionomer domains, as shown in the inset of Figure 3b.

**FIGURE 4:**
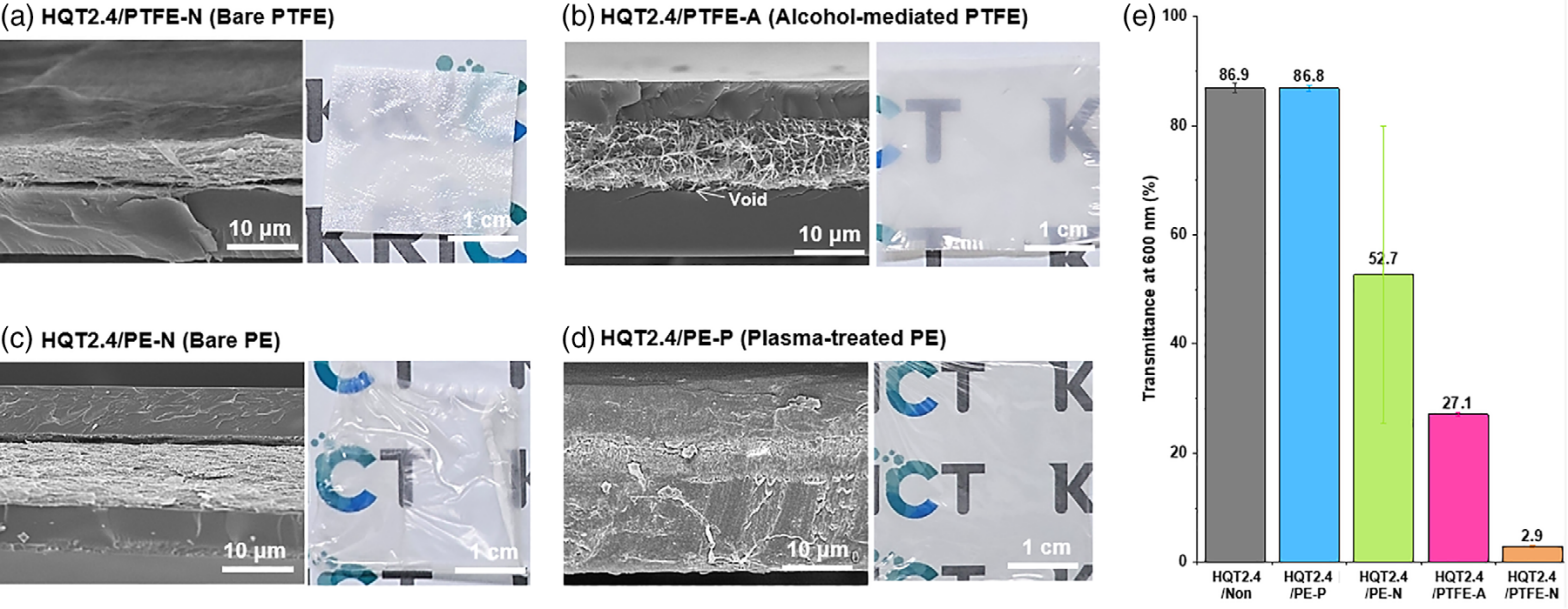
Cross‐sectional SEM and digital photographs of RCMs with different porous substrates: (a) bare porous PTFE (HQT2.4/PTFE‐N), (b) alcohol‐mediated porous PTFE (HQT2.4/PTFE‐A), (c) bare porous PE (HQT2.4/PE‐N), and (d) plasma‐treated porous PE (HQT2.4/PE‐P). (e) Transmittance at 600 nm for non‐reinforced and reinforced membranes.

As schematically summarized in Figure 3c, the impregnation behavior governed by substrate chemistry directly determines whether the RCM can simultaneously achieve low ionic resistance and effective gas tightness. Complete and continuous ionomer infiltration enables the formation of percolated ion‐conduction pathways while suppressing interfacial voids that act as gas‐transport shortcuts. In contrast, incomplete impregnation can simultaneously increase ionic resistance and hydrogen crossover by disrupting ion‐conduction pathways and leaving poorly filled regions within the porous support. The influence of substrate chemistry and impregnation behavior is clearly reflected in the morphology and optical properties of the resulting RCMs (Figure 4). For bare PTFE‐based membranes (HQT2.4/PTFE‐N), ionomer accumulation occurs primarily beneath the substrate, forming a stratified structure with extensive interfacial voids (Figure 4a). This incomplete impregnation results in pronounced optical opacity, with a transmittance of only 3% compared with 87.7% for the non‐reinforced membrane (Figure 4e and Figure S6). Alcohol‐mediated PTFE‐based membranes (HQT2.4/PTFE‐A) show modest improvement but remain highly opaque, with a transmittance of 27.3% (Figure 4b), underscoring the intrinsic limitation of PTFE substrates for hydrocarbon‐based AEMs.

In contrast, PE‐based RCMs exhibit markedly improved ionomer impregnation. Even without plasma treatment, HQT2.4/PE‐N membranes show higher transparency than HQT2.4/PTFE‐A despite lower porosity and smaller pore size (Figure 4c), reflecting improved hydrocarbon–hydrocarbon compatibility. However, pristine PE still exhibits non‐uniform impregnation, leading to thickness variation and macroscopic defects such as wrinkling (Figure S5a). This heterogeneity is reflected in large spatial variations in optical transmittance, ranging from 79.8% to 25.6% (Figure S6d). Plasma treatment eliminates these limitations. Plasma‐treated PE‐based RCMs (HQT2.4/PE‐P) exhibit complete pore filling and a uniform, void‐free composite architecture (Figure 4d), resulting in high transmittance (87.3%) comparable to that of the non‐reinforced membrane (87.7%). Asymmetric plasma treatment (HQT2.4/PE‐P‐H) further confirms the role of dual‐side plasma modification, with clear interfacial separation observed only on the untreated side (Figure S7b). To clarify the spatial extent of plasma‐induced modification within the porous PE support, additional backside XPS, cross‐sectional scanning electron microscopy–energy dispersive X‐ray spectroscopy (SEM‐EDS), wettability, and ionomer infiltration analyses were conducted (Figures S8 and S9). After one‐side treatment, the opposite (non‐exposed) surface reached an oxygen concentration of 7.31 at.% (Figure S8a) and a water contact angle of ∼43° (Figure S8b). These values are intermediate between pristine PE (1.98 at.%, ∼86°) and the directly treated surface (19.31 at.%, ∼24°), indicating that reactive oxygen species can penetrate the interconnected pore network and functionalize the support along the through‐plane direction. However, the through‐plane modification achieved by one‐side treatment remained non‐uniform and insufficient to drive complete ionomer infiltration. Cross‐sectional EDS line‐scan analysis revealed that one‐side plasma treatment produced a depth‐dependent oxygen distribution, whereas dual‐side plasma treatment resulted in a more homogeneous oxygen distribution across the PE support (Figure S8c). Consistently, ionomer‐infiltration experiments showed that one‐side plasma‐treated PE exhibited limited penetration toward the opposite surface, while dual‐side plasma‐treated PE showed substantially improved infiltration, with ionomer species observed near the opposite surface even without the second ionomer coating step (Figure S9). These results demonstrate that dual‐side plasma treatment is more effective than one‐side treatment in promoting through‐thickness wettability modification and ionomer impregnation within the dense porous PE support.

**FIGURE 5:**
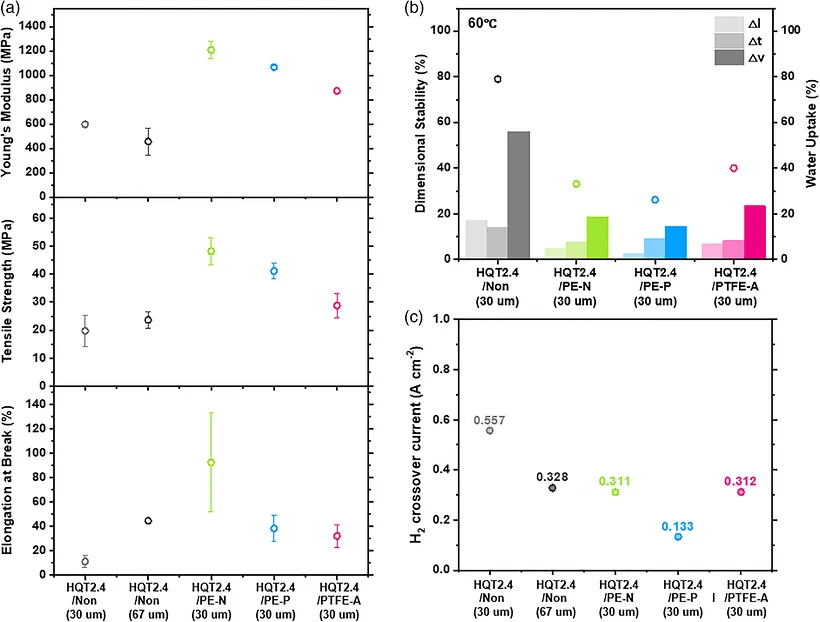
(a) Young's modulus, tensile strength, and elongation at break of 30 and 67 µm‐thick non‐reinforced membranes (HQT2.4/Non), and 30 µm‐thick RCMs (HQT2.4/PE‐N, HQT2.4/PE‐P, and HQT2.4/PTFE‐A). (b) Dimensional stability, water uptake, and (c) hydrogen crossover current densities measured at 0.6 V and 60°C of corresponding membranes.

Improved impregnation directly translates into enhanced mechanical and dimensional stability. As shown in Figure S10a,b, the porous PE substrate exhibits a tensile strength of 111.5 MPa and a Young's modulus of 941.8 MPa, corresponding to increases of 239.3% and 1358.3%, respectively, relative to porous PTFE. These superior mechanical properties are effectively transferred to PE‐reinforced membranes. Although HQT2.4/PE‐N exhibits higher apparent modulus and strength, large variations in elongation at break (Figure S10e) indicate mechanical heterogeneity arising from incomplete impregnation. Representative photographs of the tensile specimens before and after testing (Figure S11) further support this interpretation. Distinct ionomer‐impregnated and non‐impregnated regions are observed in HQT2.4/PE‐N, indicating the coexistence of mechanically dissimilar domains within the membrane. Consequently, the measured tensile response reflects contributions from both the porous PE support and the ionomer‐filled regions. Because the non‐impregnated regions retain the intrinsic mechanical characteristics of the porous PE substrate, including its high elongation at break (Figure S10a), HQT2.4/PE‐N exhibits increased specimen‐to‐specimen variation (Figure 5a) and a higher apparent elongation at break compared with the fully impregnated HQT2.4/PE‐P membrane.

In contrast, HQT2.4/PE‐P shows uniform mechanical behavior consistent with a fully impregnated composite structure, indicating effective load transfer between the ionomer matrix and the PE reinforcement. This homogeneous mechanical response is accompanied by markedly improved dimensional stability, as confirmed by measurements at 60°C, where HQT2.4/PE‐P exhibits the lowest dimensional change and water uptake among all tested membranes (Figure 5b, Figure S12, and Table 1). These results demonstrate that complete impregnation of the PE substrate not only suppresses mechanical heterogeneity but also constrains hydration‐induced swelling.

**TABLE 1:** Physicochemical, mechanical, and ionic transport properties of the tested AEMs.

AEMs	Dry Thickness [µm]	Young's Modulus [MPa]	Tensile Strength [MPa]	Elongation at Break [%]	Dimensional Change (at 60°C)			Water Uptake (at 60°C) [%]	Estimated Effective Through‐Plane Ionic Conductivity^a^ [mS cm^−1^]
					Δl [%]	Δt [%]	Δv [%]		
HQT2.4/Non	30	598.3 ± 25.5	18.8 ± 5.5	11.1 ± 4.9	16.9	14.1	56.0	79.0	160.2
	67	456.9 ± 110.8	23.7 ± 2.9	44.6 ± 17.2	−	−	−	−	160.2
HQT2.4/PE‐N	30	1207.6 ± 71.8	48.2 ± 4.8	92.5 ± 40.5	5	7.7	18.7	33.1	90.3
HQT2.4/PE‐P	30	1066.7 ± 15.3	41.1 ± 2.7	38.3 ± 10.7	2.5	9.0	14.5	26.2	108.2
HQT2.4/PTFE‐A	30	872.3 ± 23.6	28.8 ± 4.3	32.1 ± 9.1	6.8	8.3	23.5	40.0	115.3

^a^ Estimated from the HFR measured at 3.0 A cm^−^^2^ under 1.0 M KOH feed at 60°C after subtracting the non‐membrane resistance (12.7 mΩ cm^2^) obtained from thickness‐dependent extrapolation of the non‐reinforced HQT2.4 membrane series. Identical electrodes, cell hardware, and assembly procedures were used to minimize variations in non‐membrane resistance. The values are resistance‐derived estimates, as membrane‐specific variations in contact resistance associated with hydration and swelling cannot be completely excluded (see Note S1).

Hydrogen crossover measurements (Figure 5c and Figure S13) reveal the critical advantage of plasma‐treated PE‐based RCMs. HQT2.4/PE‐P exhibits a crossover current density of 0.133 mA cm^−2^ at 0.6 V, representing more than a fourfold reduction compared with the non‐reinforced membrane of the same thickness (30 µm, 0.557 mA cm^−2^). Its gas‐barrier performance also surpasses that of a much thicker non‐reinforced membrane (67 µm, 0.328 mA cm^−2^) and alcohol‐mediated PTFE‐based RCMs (0.312 mA cm^−2^). This improvement originates from complete ionomer filling of the dense PE pore network, which suppresses long‐range void pathways and forms a compact diffusion barrier. While HQT2.4/PE‐N also reduces hydrogen crossover relative to the non‐reinforced membrane, residual voids result in inferior barrier performance compared with HQT2.4/PE‐P.

### 2.3 Electrochemical Performance and Durability of RCMs

The practical applicability of the prepared RCMs in water electrolysis was evaluated and compared with that of non‐reinforced membranes. RCM‐based AEMWEs were assembled using Ni_2_Fe nanofoam (1.0 mg_NiFe_ cm^−2^) as the anode catalyst and PtRu/C (0.5 mg_PtRu_ cm^−2^) as the cathode catalyst, and all measurements were conducted in 1.0 m KOH at 60°C.

**FIGURE 6:**
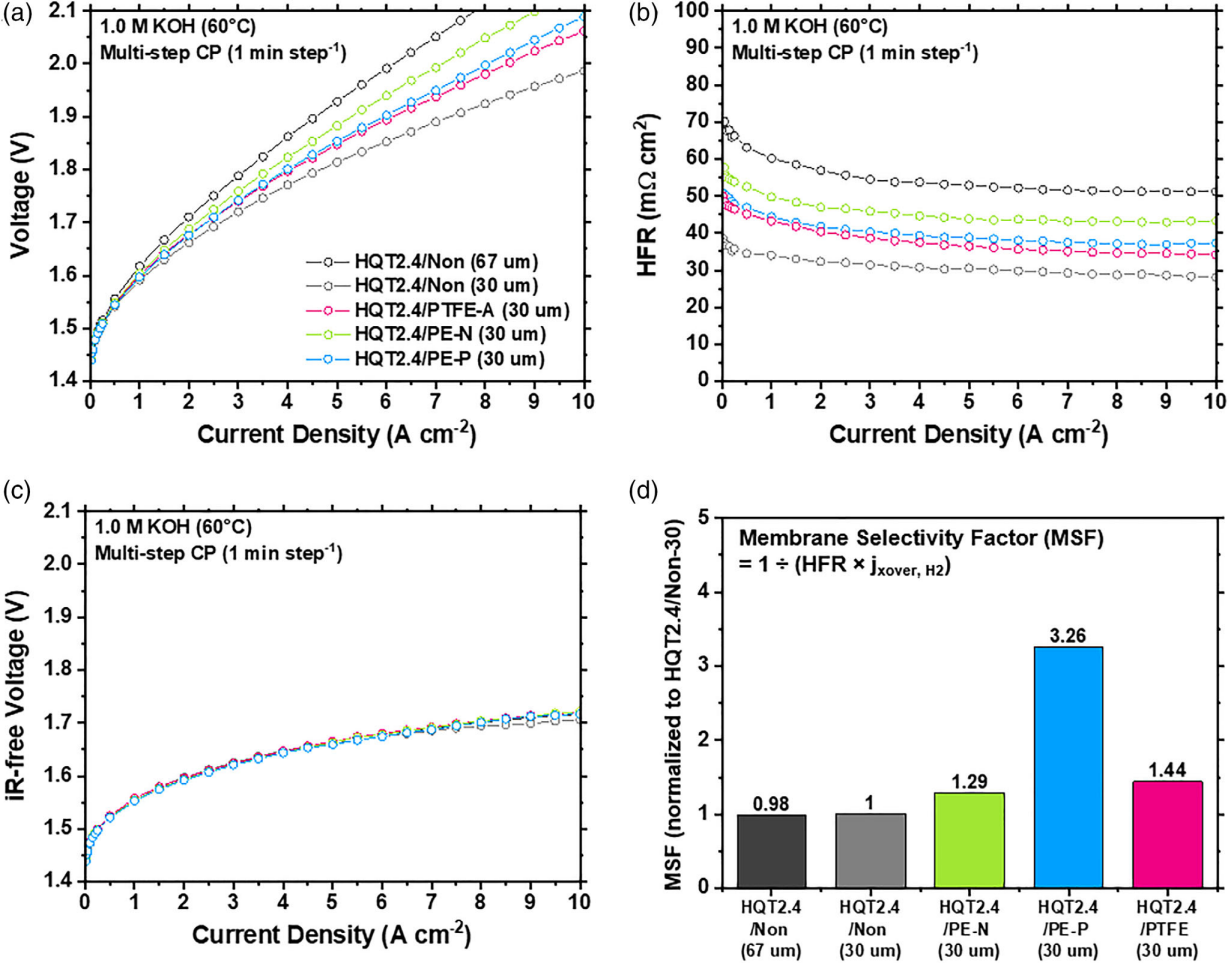
(a) Polarization curves measured under 1.0 m KOH at 60°C using a multi‐step chronopotentiometry for non‐reinforced and reinforced membranes. Corresponding (b) high‐frequency resistance (HFR) and (c) iR‐free voltage extracted from electrochemical impedance spectroscopy measured at each current step. (d) Membrane selectivity factor (MSF), defined as 1/(HFR × j_H2, crossover_), normalized to the MSF value of 30 µm non‐reinforced membrane (HQT2.4/Non, 30 µm).

As shown in Figure 6a, all 30 µm‐thick RCMs exhibited slightly inferior performance compared with the 30 µm‐thick non‐reinforced membrane. This behavior is inherent to RCM architectures, as the porous reinforcement substrates are ionically insulating and dilute the effective ion‐conducting phase within the membrane. Nevertheless, the primary advantage of reinforcement lies in its ability to enhance mechanical stability and suppress hydrogen crossover (Figure 5), thereby enabling the use of thinner membranes without compromising operational safety. This benefit is clearly demonstrated by comparison with the thicker non‐reinforced membrane (67 µm). Despite exhibiting hydrogen crossover levels comparable to or even lower than that of the 67 µm non‐reinforced membrane (Figure 5c), all RCMs delivered significantly higher electrochemical performance. In particular, the alcohol‐mediated PTFE‐reinforced membrane (HQT2.4/PTFE‐A) achieved a cell voltage of 1.740 V with an HFR of 38.7 mΩ cm^2^ at 3.0 A cm^−^^2^, outperforming the thick non‐reinforced membrane (1.788 V, 54.5 mΩ cm^2^). Although the PTFE substrate was not fully impregnated with HQT2.4 ionomer, the reduced membrane thickness (∼30 µm), together with substantial swelling under 1.0 m KOH feeding conditions, likely contributed to the lowered ohmic resistance. Ionic uptake expands ionomer domains and partially collapses residual void spaces, while the large pore size and high porosity of PTFE facilitate the formation of continuous ionic pathways. However, such swelling‐induced performance gains should be interpreted with caution, as excessive swelling is closely associated with compromised dimensional stability and increased hydrogen crossover, highlighting that performance metrics alone are insufficient for evaluating membrane suitability.

Meanwhile, the PE‐reinforced membrane without plasma treatment (HQT2.4/PE‐N) exhibited inferior performance, with a higher cell voltage (1.759 V) and larger HFR (45.9 mΩ cm^2^) at 3.0 A cm^−^^2^ compared with HQT2.4/PTFE‐A. This behavior is attributed to the intrinsically dense pore network of the PE substrate (Figure 2), which limits absolute ionomer uptake despite favorable substrate–ionomer affinity (Figure 1). Notably, Plasma modification effectively eliminates this limitation. The plasma‐treated PE‐reinforced membrane (HQT2.4/PE‐P) exhibited a significantly reduced HFR of 40.4 mΩ cm^2^ and a corresponding cell voltage of 1.742 V at 3.0 A cm^−^^2^, approaching the performance of the optimized PTFE‐based RCM, despite the smaller pore size and lower porosity of the PE substrate. Importantly, the PE‐based membrane simultaneously provides superior mechanical stability and markedly enhanced gas tightness relative to PTFE‐based RCMs. Because the dense pore morphology of PE is preserved during diffusion‐dominated oxygen plasma treatment, the observed performance improvement can be attributed to maximized ionomer impregnation enabled by plasma‐induced surface functionalization. The single‐side plasma‐treated membrane (HQT2.4/PE‐P‐H) exhibited intermediate performance (1.755 V, 43.8 mΩ cm^2^), directly confirming that plasma modification governs the degree of effective ionomer impregnation and ionic conduction (Figure S14). To further quantify the membrane‐level ionic transport behavior, the effective through‐plane ionic conductivity was estimated by correcting the measured resistance using the non‐membrane contribution obtained from thickness‐dependent extrapolation of the HQT2.4 membrane series (Figure S15). The estimated values are also summarized in Table 1 together with the mechanical properties, dimensional changes, and water uptake for direct comparison. The non‐reinforced HQT2.4 membrane exhibited an effective through‐plane ionic conductivity of approximately 160.2 mS cm^−1^, while the estimated values for HQT2.4/PE‐N, HQT2.4/PE‐P, and HQT2.4/PTFE‐A were 90.3, 108.2, and 115.3 mS cm^−1^, respectively. The higher conductivity of HQT2.4/PE‐P compared with HQT2.4/PE‐N further supports the improved through‐plane ionic transport achieved by plasma‐assisted ionomer impregnation. All MEAs were fabricated using identical electrodes, cell hardware, and assembly procedures to minimize variations in non‐membrane resistance. However, membrane‐specific differences in hydration and swelling may affect membrane–electrode contact resistance; therefore, these values are regarded as resistance‐derived estimates rather than independently measured intrinsic membrane conductivities. The detailed calculation, assumptions, and limitations are provided in Note S1. Notably, the iR‐free voltages of all MEAs remained nearly identical (∼1.62 V at 3.0 A cm^−^^2^; Figure 6c and Figure S14c), indicating that intrinsic electrode kinetics and mass‐transport characteristics were unaffected by membrane reinforcement. Therefore, the observed differences in cell voltage primarily originate from membrane ohmic losses.

From these results, it becomes evident that membrane performance in electrolysis is governed not by ionic resistance or hydrogen crossover alone, but by their coupled balance. To provide a cell‐relevant comparison of this balance, we defined a membrane selectivity factor (MSF) as:

$$\mathrm{MSF}\;=\frac{1}{HFR\times j_{H2,Crossover}}$$

where HFR is the high‐frequency resistance measured at 3.0 A cm^−2^ in the assembled cell under 1.0 m KOH‐fed operation at 60°C (Figure 6b), and j_H2, crossover_ is the hydrogen crossover current density measured by linear sweep voltammetry (LSV) at 0.6 V under fully humidified H_2_/N_2_‐fed condition at 60°C (Figure 5c). The corresponding HFR, j_H2, crossover_, and normalized MSF values are summarized in Table 2. Under the specified measurement conditions, a higher MSF indicates a more favorable balance between cell‐level ohmic resistance and hydrogen crossover. The physical rationale of the MSF can be understood from the opposite thickness dependence of ohmic resistance and gas crossover. The MSF values were normalized to that of the 30‐µm non‐reinforced membrane (HQT2.4/Non, 30 µm). The two non‐reinforced membranes of different thickness (30 and 67 µm) exhibited comparable normalized MSF values despite their substantially different HFR and hydrogen crossover values, consistent with the expectation that the MSF partially compensates for geometric thickness effects and therefore primarily reflects the membrane's selectivity between ion transport and hydrogen permeation (Note S2). To corroborate this interpretation, commercial PiperION‐A membranes with different thicknesses (21, 40, and 62 µm) were also evaluated under the same cell configuration and test protocol (Figures S13b and S16). The normalized MSF values remained within a similar range of 0.9–1.1, supporting the expectation that membranes of fixed chemistry exhibit comparable MSF values across the tested thickness range under an identical measurement protocol. In contrast, all reinforced membranes deviated markedly from this baseline behavior, indicating that reinforcement substantially shifts the membrane selectivity. As shown in Figure 6d, the plasma‐treated PE‐reinforced membrane (HQT2.4/PE‐P) achieved the highest normalized MSF value of 3.26, corresponding to 3.26‐fold and 2.26‐fold enhancements compared with the non‐reinforced membrane (HQT2.4/Non) and the optimized PTFE‐based RCM (HQT2.4/PTFE‐A), respectively. The PE‐reinforced membrane without plasma treatment (HQT2.4/PE‐N) exhibited a lower MSF than the PTFE‐based RCM, highlighting the critical role of plasma‐induced ionomer impregnation in unlocking the performance potential of dense PE substrates.

**TABLE 2:** Hydrogen crossover current density (j_H2, crossover_), high frequency resistance (HFR), and normalized membrane selectivity factor (MSF) values of the membranes.

AEMs	Dry Thickness [µm]	j_H2, crossover_ ^a^ [mA cm^−2^]	HFR^b^ [mΩ cm^2^]	MSF^c^ [µm]
HQT2.4/Non	30	0.557	31.4	1.00
	67	0.327	54.5	0.98
HQT2.4/PE‐N	30	0.311	45.9	1.23
HQT2.4/PE‐P	30	0.133	40.4	3.26
HQT2.4/PTFE‐A	30	0.312	38.7	1.44
PiperION‐A	21	0.588	33.4	0.89
	40	0.368	50.8	0.94
	62	0.235	69.1	1.08

^a^ Hydrogen crossover current density measured by linear sweep voltammetry at 0.6 V under fully humidified H_2_/N_2_ condition at 60°C.

^b^ High‐frequency resistance measured at 3.0 A cm^−2^ under 1.0 m KOH feed condition at 60°C.

^c^ Values were normalized to that of the 30‐µm non‐reinforced HQT2.4 membrane.

**FIGURE 7:**
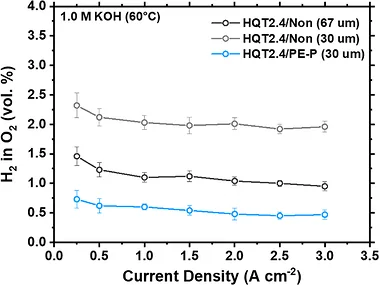
Hydrogen concentration in the anode outlet during electrolysis under 1.0 m KOH at 60°C. The red dashed line denotes the 50% of low explosion limit.

The low gas crossover through the plasma‐treated PE‐based RCM was further evaluated under initial electrolyzer operating conditions by monitoring hydrogen concentration in the anode outlet (H_2_ in O_2_) during electrolysis (Figure 7). The 30‐µm non‐reinforced membrane exhibited a markedly higher anodic hydrogen concentration across a wide current density range, indicating insufficient gas‐barrier capability at reduced thickness. In contrast, the HQT2.4/PE‐P membrane maintained a consistently low hydrogen concentration of 0.5–0.75 vol.%, which is nearly half that of the much thicker 67‐µm non‐reinforced membrane. This behavior is consistent with its lower hydrogen crossover current density obtained from LSV measurements (Figure 5c), confirming that complete ionomer impregnation within the dense PE pore network effectively suppresses hydrogen crossover under the tested electrolysis conditions.

**FIGURE 8:**
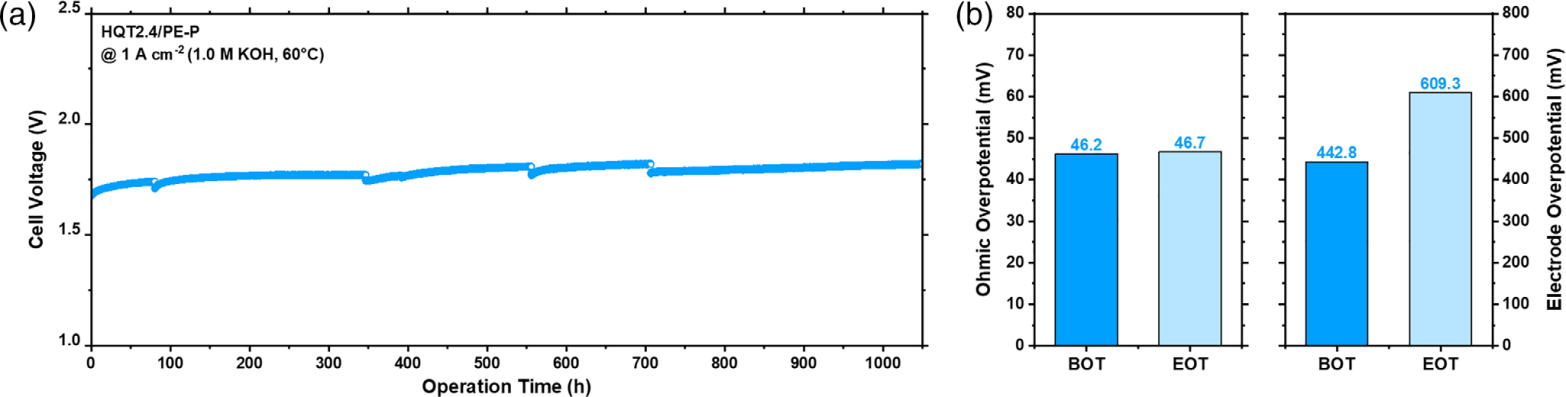
(a) Long‐term durability test of HQT2.4/PE‐P‐based AEMWE conducted at a constant current density of 1.0 A cm^−2^ under 1.0 m KOH at 60°C for over 1000 h. (b) Comparison of ohmic overpotential and total electrode overpotential at the beginning of test (BOT) and end of test (EOT).

The long‐term durability of the plasma‐treated PE‐based RCM was evaluated at a constant current density of 1.0 A cm^−^^2^ in 1.0 m KOH at 60°C under a dry‐cathode configuration (Figure 8a). Stable cell operation was maintained for over 1000 h under prolonged electrolysis. Although minor voltage fluctuations were observed due to temporary system shutdowns, the high‐frequency resistance remained nearly unchanged, increasing by only ∼1% after long‐term operation (Figure S17). This result indicates that no substantial increase in cell‐level ohmic resistance occurred during the durability test. The stable operation and nearly unchanged HFR under prolonged dry‐cathode conditions suggest that membrane‐mediated water transport was sufficient to support the required water balance. To further evaluate the stability of the RCM after the durability test, post‐mortem analyses were conducted on the membrane recovered after the durability test (Figure S18). The recovered membrane exhibited a hydrogen crossover current density of 0.262 mA cm^−2^ at 0.6 V (Figure S19a). Although this value was higher than that of the pristine HQT2.4/PE‐P membrane (0.133 mA cm^−2^), it remained 20.1% lower than that of the substantially thicker pristine non‐reinforced 67 µm HQT2.4/Non membrane (0.328 mA cm^−2^). This result indicates that the reinforced membrane retained effective gas‐barrier properties after long‐term operation. In addition, cross‐sectional SEM revealed an average membrane thickness of approximately 27.5 µm after durability testing (Figure S19b), indicating that substantial membrane thinning did not occur during the durability test. Together with the minimal HFR increase observed during operation, these post‐mortem results suggest that the RCM retained its gas‐barrier properties and structural integrity during prolonged electrolysis, and that membrane degradation was not the dominant origin of the observed performance decay. Instead, the voltage increase is more reasonably attributed to electrode degradation. For instance, TEM images of the cathode catalyst (Figure S20) show significant Pt nanoparticle agglomeration after durability testing, consistent with the observed increase in electrode overpotential (Figure 8b). Overall, these results support the long‐term functional stability of the HQT2.4/PE‐P RCM under the tested AEMWE conditions.

## 3 Conclusion

In this study, we demonstrate that plasma‐modified porous PE serves as an effective reinforcement platform for hydrocarbon‐based anion exchange membranes, enabling the fabrication of thin, mechanically robust RCMs with low ionic resistance and low hydrogen crossover for AEMWE. Unlike conventional ePTFE reinforcements, which suffer from severe fluorocarbon–hydrocarbon interfacial incompatibility and incomplete ionomer impregnation, oxygen plasma treatment renders the PE surface highly wettable and chemically compatible, enabling homogeneous infiltration of the hydrocarbon ionomer throughout its dense submicron pore network. As a result, the plasma‐treated PE‐based RCM achieves mechanical strength, suppressed swelling, and substantially lower hydrogen crossover compared with non‐reinforced membranes and optimized ePTFE‐based RCMs. Notably, despite its thin thickness of only 30 µm, the PE‐based RCM maintained H_2_‐in‐O_2_ concentrations below the safety limit under initial electrolysis operation. Electrochemical evaluation further reveals that plasma‐induced ionomer impregnation effectively mitigates the ohmic resistance penalty associated with the dense PE pore architecture, allowing the PE‐based RCM to achieve AEMWE performance comparable to optimized PTFE‐based systems.

To quantitatively capture the coupled balance between ionic resistance and gas crossover, the MSF was employed as a unified performance metric. The plasma‐treated PE‐based RCM exhibits the highest MSF among all tested membranes, exceeding those of the non‐reinforced and ePTFE‐reinforced membranes by factors of 3.26 and 2.26, respectively. These results suggest that plasma‐engineered PE reinforcement is an effective strategy for improving the balance between ionic resistance and hydrogen crossover in hydrocarbon‐based AEMs. Long‐term testing showed only a minor HFR increase over 1000 h, while post‐mortem analyses indicated retention of membrane thickness, structural integrity, and effective gas‐barrier functionality. The observed performance decay was predominantly associated with electrode degradation. Importantly, the plasma‐functionalization strategy developed in this work is compatible with atmospheric‐pressure plasma technologies widely employed in continuous roll‐to‐roll processing of polymer films, providing a practical pathway toward scalable membrane manufacturing. These results establish plasma‐modified porous PE as a viable reinforcement platform for next‐generation hydrocarbon‐based AEMs, offering a practical pathway toward high‐efficiency, durable, and safe AEM water electrolysis.

## 4 Experimental Section

### 4.1 Materials

Pt/C (40 wt.%) and PtRu/C (40 wt.% Pt, 20 wt.% Ru) catalysts were purchased from Alpha Aesar. Stainless steel felt (SUS316, ∼300 µm) and microporous layer–coated gas diffusion layer (GDL, JNT30‐A3, ∼315 µm) were supplied by Bekaert Inc. and JNTG Inc., respectively. Porous polyethylene (PE, 9 µm) and porous polytetrafluoroethylene (PTFE, 7 µm) sheets were obtained from DEEPSMARTECH Inc. and SYNOPEX Co., Ltd. All solvents and KOH (85 wt.%) were purchased from Samchun Chemical and used as received.

### 4.2 Fabrication of Non‐Reinforced Membrane and Ionomer Solutions

The high‐molecular‐weight quaternized polycarbazole anion conducting polymer used in this study, hereafter denoted as HQT2.4 (Mw = 130 kDa), was synthesized following previously reported procedures (Figure S21) [29]. HQT2.4 refers to quaternized poly(9‐(6‐(trimethylammonium bromide)hexyl)‐9H‐carbazole‐co‐1,1,1‐trifluoroisopropane‐co‐9‐hexyl‐9H‐carbazole with ion exchange capacity of 2.4 meq g^−1^. Non‐reinforced membranes with dry thicknesses of 30 and 67 µm were prepared by solvent casting from a deionized water/isopropanol/N‐methyl‐2‐pyrrolidone (2:2:1, w/w) solution (15 wt.%). Membranes were converted to the hydroxide form by immersion in 1.0 m KOH, followed by rinsing and water equilibration. Ionomer solutions were prepared by dispersing HQT2.4 in an H_2_O/n‐propanol mixture (6:4, w/w).

### 4.3 Preparation of PTFE‐Based Reinforced Composite Membranes

PTFE‐reinforced membranes were fabricated using an alcohol‐mediated impregnation technique [22]. Briefly, a porous PTFE sheet was placed onto a gelated HQT2.4 ionomer layer, followed by sequential bar coating of ethanol and ionomer solution using a dual‐bar coating process (coating speed: 4 mm s^−^^1^; bar gap: 280 µm), where the ethanol residual time was controlled to ∼70 s to temporarily alleviate PTFE non‐wettability. Alcohol‐treated (HQT2.4/PTFE‐A) and untreated (HQT2.4/PTFE‐N) membranes with final dry thicknesses of ∼30 µm were obtained after thermal drying at 80°C and 100°C.

### 4.4 Preparation of PE‐Based Reinforced Composite Membranes

Porous PE sheets were treated by oxygen plasma for 2 min (O_2_ flow: 10 sccm, RF power: 25 W, pressure: ∼3.2 × 10^−^^1^ Torr) using a CCP system operated in plasma mode. To achieve uniform functionalization throughout the porous PE support, both sides of the PE sheet were sequentially exposed to oxygen plasma under identical conditions (dual‐side plasma treatment). Detailed operating principles of the plasma mode and processing parameters are provided in Figure S3 and Table S1. Plasma‐treated or pristine PE sheets were subsequently integrated into composite membranes by sequential bar coating of HQT2.4 ionomer solution, enabling capillary‐driven impregnation into the porous PE matrix. Following plasma treatment, the PE sheet was carefully placed onto a gelated HQT2.4 ionomer layer cast on a glass substrate and subsequently covered by an additional ionomer coating via bar coating, enabling uniform infiltration throughout the porous PE matrix. The membranes were dried at 80°C and 100°C to obtain reinforced composite membranes with final dry thicknesses of ∼30 µm.

### 4.5 Characterizations of Porous Substrates and Membranes

Morphologies were analyzed by field‐emission SEM (SU‐5000, Hitachi). Pore‐size distributions were measured by mercury intrusion porosimetry (0.003–1000 µm, AutoPore V9600). Because the porous substrates used in this study were very thin, approximately 7 µm for ePTFE and 9 µm for PE, multiple sheets were cut and stacked to satisfy the minimum sample mass (0.5 g) required for MIP analysis. Chemical compositions were characterized by XPS (ESCALAB250), and surface wettability was evaluated by static water contact‐angle measurements (5 µL droplets, 25°C). Mechanical properties were measured in the dry state by uniaxial tensile testing (5 mm min^−^^1^). Optical transmittance was measured by UV–vis spectroscopy (300–800 nm). Water uptake and dimensional changes were evaluated after immersion in water at 80°C for 12 h. Hydrogen crossover was evaluated by linear sweep voltammetry using Pt/C‐coated GDL electrodes (0.2 mg_Pt_ cm^−2^) assembled on both sides of the membrane in a single‐cell configuration. Fully humidified H_2_ and N_2_ were supplied to the anode and cathode, respectively, at 0.2 L min^−^^1^ and 60°C. The H_2_‐fed Pt/C electrode served as both the counter and pseudo‐reference electrode, while the N_2_‐fed Pt/C electrode functioned as the working electrode. LSV was performed from 0 to 0.6 V at 2 mV s^−2^, and the hydrogen crossover current density was determined from the hydrogen oxidation current measured at 0.6 V.

### 4.6 MEA Fabrication and Single‐Cell Assembly

Membranes were converted to the hydroxide form by immersion in 1.0 m KOH for 3 h. Membrane electrode assemblies (MEAs) were fabricated using the catalyst‐coated substrate method. The catalyst‐coated area was defined as 2.23 cm × 2.24 cm, corresponding to an active area of 5 cm^2^. For the anode, Ni_2_Fe nanofoam catalyst, prepared according to previously reported methods [31, 32], was dispersed in deionized water and 2‐propanol, followed by the addition of HQT2.4 ionomer to achieve an ionomer‐to‐catalyst ratio of 10 wt.%. The resulting ink was sprayed onto stainless steel felt (10AL3) to obtain a catalyst loading of 1.0 mgNiFe cm^−^^2^. For the cathode, PtRu/C catalyst ink was prepared with an ionomer‐to‐catalyst ratio of 20 wt.% and sprayed onto carbon paper (SGL‐39BC) to achieve a loading of 0.4 mgPtRu cm^−^^2^. Single cells were assembled using SUS‐316L end plates, Au‐coated current collectors, and Pt‐coated Ti flow plates with a single serpentine channel (1 mm width × 1 mm height). PTFE gaskets with thicknesses of 270 µm (anode) and 220 µm (cathode) were used to complete the cell assembly.

### 4.7 AEMWE Performance and Durability Measurements

The AEMWE performance was evaluated at 60°C under symmetric 1.0 m KOH‐fed conditions, with the electrolyte preheated to the cell temperature. Polarization curves were obtained by multistep chronopotentiometry, holding each current density for ≥1 min. Measurements were conducted over a current density range of 0.02–10.0 A cm^−^^2^ using 27 predefined current steps for 5 cm^2^ single cells. Electrochemical impedance spectroscopy (EIS) was performed at each current density over a frequency range of 1 Hz to 100 kHz with an AC amplitude of 8% of the applied current. All electrochemical performance measurements, including electrochemical impedance spectroscopy (EIS), were conducted using a BioLogic HCP‐803 potentiostat. Hydrogen crossover during electrolysis was quantified by measuring the hydrogen concentration in the anode outlet (H_2_ in O_2_) using a thermal conductivity detector (TCD)‐based hydrogen analyzer (FCT 320). Before reaching the detector, the anode outlet gas was passed through a chiller and a dryer to remove entrained moisture, with the condensate drained. The TCD was calibrated with H_2_/N_2_ standard mixtures containing 0.05, 0.1, 1.0, and 2.5 vol.% H_2_. H_2_/N_2_ mixtures were used instead of H_2_/O_2_ mixtures to avoid the explosion hazard associated with handling H_2_/O_2_ gas mixtures. Because the thermal conductivity of O_2_ (∼26.5 mW m^−1^ K^−1^) is slightly higher than that of N_2_ (∼26 mW m^−1^ K^−1^), the N_2_‐based calibration introduces a small positive offset in the measured H_2_ concentration when the balance gas is O_2_. This offset was evaluated by supplying humidified O_2_ (with 60°C dew point) through the anode‐vent line to the analyzer, which yielded an apparent reading of 0.34 vol.% H_2_. The offset was not subtracted from the reported values, which therefore represent conservative upper‐bound estimates of the H_2_‐in‐O_2_ concentrations. Cells were operated under steady‐state conditions at 60°C with continuous circulation of 1.0 m KOH, and the anode outlet stream was periodically sampled for analysis. Durability tests were conducted at a constant current density of 1.0 A cm^−^^2^ and 60°C for over 1000 h under asymmetric feeding conditions (dry cathode) using a WonATech MP10 potentiostat. In the event of unplanned shutdowns, polarization curves were re‐recorded before resuming the durability test. For durability measurements, a Pt/C cathode catalyst with a loading of 0.5 mg_Pt_ cm^−^^2^ was used.

## Author Contributions

**Sungjun Kim**: methodology, writing – original draft, visualization, supervision, funding acquisition; **Yeram Shin**: formal analysis, investigation, data curation; **Minseop So**: resources, formal analysis; **Jungwon An**: data curation; **Dong‐Chan Kim**: resources; **Jang Yong Lee**: resource, writing – review & editing, funding acquisition; **Segeun Jang**: conceptualizaion, methodology, writing – original draft, writing – review & editing, visualization, supervision, project administration, funding acquisition.

## Funding

This research was supported by the Open R&D Program of Korea Electric Power Corporation (Grant No. R23XO04), Nano & Material Technology Development Program through the National Research Foundation of Korea (NRF), funded by the Ministry of Science and ICT (Grant No. RS‐2024‐00409675), National Research Foundation of Korea (NRF) grant funded by the Korea government (MSIT) (Grant No. RS‐2024‐00467234), and Korea Research Institute of Chemical Technology (KRICT) (KS2622‐20).

## Conflicts of Interest

The authors declare no conflicts of interest.

## Supporting information




**Supporting File**: advs77467‐sup‐0001‐SuppMat.docx.

## Data Availability

The data that support the findings of this study are available from the corresponding author upon reasonable request.
