# Supplementary material for: Plasma‐Engineered Polyethylene‐Reinforced Anion Exchange Membranes With Low Ionic Resistance and Low Hydrogen Crossover for Water Electrolysis
Source: Adv Sci (Weinh). 2026 Aug 29:e77467. Online ahead of print. doi: 10.1002/advs.77467 (PMC13525615; doi:10.1002/advs.77467)
Supplement: Supplementary file 1 [file ADVS-9999-e77467-s001.docx]

Supporting Information

**Plasma-Engineered Polyethylene-Reinforced Anion Exchange Membranes with Low Ionic Resistance and Low Hydrogen Crossover for Water Electrolysis**

Sungjun Kim, Yeram Shin, Minseop So, Jungwon An, Dong-Chan Kim, Jang Yong Lee^*^, Segeun Jang^*^

S. Kim, Y. Shin

Hydrogen Energy Research Center, Korea Research Institute of Chemical Technology (KRICT), Daejeon 34114, Republic of Korea

M. So, J. An, S. Jang

School of Mechanical Engineering, Kookmin University, Seoul 02707, Republic of Korea
E-mail: sjang@kookmin.ac.kr

D.-C. Kim, J. Y. Lee

Department of Chemical Engineering, Konkuk University, Seoul 05029, Republic of Korea

E-mail: [ljyljy78@konkuk.ac.kr](mailto:ljyljy78@konkuk.ac.kr)

S. Kim and Y. Shin contributed equally to this work.

**Funding:**

Open R&D program of Korea Electric Power Corporation (Grant No. R23XO04).

Nano & Material Technology Development Program through the National Research Foundation of Korea (NRF), funded by the Ministry of Science and ICT (Grant No. RS-2024-00409675).

National Research Foundation of Korea (NRF) grant funded by the Korea government (MSIT) (Grant No. RS-2024-00467234).

**Keywords:** Anion exchange membrane water electrolysis (AEMWE), Porous polyethylene (PE), Porous polytetrafluoroethylene (PTFE), Plasma treatment, Impregnation, Gas tightness

**Table of contents**

**Supplementary Notes	4**

Note S1	4

Note S2	5

**Supplementary Figures	7**

Figure S1	7

Figure S2	8

Figure S3	9

Figure S4	10

Figure S5	11

Figure S6	12

Figure S7	13

Figure S8	14

Figure S9	15

Figure S10	16

Figure S11	17

Figure S12	18

Figure S13	19

Figure S14	20

Figure S15	21

Figure S16	22

Figure S17	23

Figure S18	24

Figure S19	25

Figure S20	26

Figure S21	27

**Supplementary Tables	28**

Table S1	28

**Supplementary Notes**

**Note S1. Estimation and limitation of effective through-plane ionic conductivity of RCMs**

The HFR measured in the assembled cell contains both membrane resistance and non-membrane contributions, including membrane–electrode contact and hardware electrical resistances. To estimate the effective through-plane ionic conductivity, the non-membrane contribution was determined from the thickness dependence of HFR for the non-reinforced HQT2.4 membrane series measured under an identical cell configuration. Linear extrapolation to zero membrane thickness yielded a non-membrane resistance of approximately 12.7 mΩ cm^2^ (**Figure S15**).

This non-membrane resistance was used as a first-order correction for the HQT2.4-based membranes measured using identical electrodes, porous transport layers, flow fields, and assembly procedures to minimize variations in non-membrane resistance. After correction, the effective through-plane ionic conductivities were estimated to be approximately 160.2 mS cm^-1^ for the non-reinforced HQT2.4 membrane, 90.3 mS cm^-1^ for HQT2.4/PE-N, 108.2 mS cm^-1^ for HQT2.4/PE-P, and 115.3 mS cm^-1^ for HQT2.4/PTFE-A.

These values should be regarded as resistance-derived estimates rather than independently measured intrinsic membrane conductivities. Rigorous membrane-specific correction would require separate thickness-dependent extrapolation for each RCM while proportionally maintaining its three-layer architecture (non-reinforced skin/reinforced region/non-reinforced skin), reinforcement characteristics, and degree of ionomer impregnation, which is difficult to ensure experimentally. Therefore, the reported values represent estimated effective through-plane ionic conductivities under the stated common non-membrane resistance assumption.

**Note S2. Physical basis, structural dependence, and scope of the membrane selectivity factor (MSF)**

The membrane selectivity factor (MSF) was introduced to provide a practical comparison of the coupled trade-off between ohmic resistance and hydrogen crossover in membrane-based electrolysis. In this study, the MSF is defined as:

$$MSF= \frac{1}{HFR\times j_{H2, Crossover}}$$

where HFR is the high-frequency resistance measured in the assembled cell and j_H₂,crossover_ is the hydrogen crossover current density. A higher MSF indicates that a membrane configuration can simultaneously reduce cell-level ohmic resistance and hydrogen crossover. Therefore, the MSF is intended to compare the balance between ion-transport resistance and gas-barrier performance, rather than to evaluate either property independently.

The physical rationale of the MSF can be understood from an ideal membrane-dominated case. If the measured HFR is governed primarily by the membrane ohmic resistance, it can be approximated as HFR ≈ l/σ. In addition, if the measured hydrogen crossover current is not affected by parasitic currents, such as charging or shorting currents, it can be approximated as j_H2,crossover_ ≈ 2F·P_H2_·Δp/l. Under this idealized case, the MSF can be expressed as:

$$MSF (ideal case)= \frac{\sigma}{2F\times\Delta p\times P_{H2}}$$

where *l* is the membrane thickness, *σ* is the ionic conductivity, *F* is the Faraday constant, *P_H2_* is the hydrogen permeability of the membrane, and *Δp* is the hydrogen partial-pressure difference across the membrane. In this ideal case, the membrane thickness term cancels out, and the MSF is governed by the intrinsic conductivity-to-permeability ratio, σ/P_H2_. This provides the rationale for using the MSF as a single comparative descriptor of the coupled balance between ohmic resistance and hydrogen crossover.

Importantly, although the geometric thickness contribution cancels out under this idealized assumption, intrinsic membrane properties, such as polymer chemistry and ion-exchange capacity (IEC), still influence the MSF by changing the transport parameters themselves. For example, a higher IEC can lower ohmic resistance by increasing the density of ion-conducting groups and enhancing hydroxide transport. However, excessive IEC can also increase water uptake and swelling, which may raise hydrogen permeability. Therefore, the MSF is designed to reduce the direct influence of simple geometric thickness effects while reflecting intrinsic membrane characteristics that govern the ion-transport/gas-permeability balance.

It should also be noted that the idealized thickness cancellation is not exact in practical cell measurements. HFR can include not only membrane ionic resistance but also other cell-level ohmic contributions, such as electronic/contact resistance and interfacial resistance. Similarly, the measured hydrogen crossover current density can be influenced not only by hydrogen permeation through the membrane but also by measurement conditions and parasitic current contributions, such as capacitive charging or internal shorting currents. Therefore, the MSF should not be interpreted as a purely intrinsic material constant. Instead, it should be regarded as a condition-specific comparative descriptor based on cell-level measurements that can partially compensate for simple geometric thickness effects and is most meaningful when membranes are evaluated under the same cell configuration and measurement protocol.

**Supplementary Figures**

**Figure S1.** Contact angles of DI water and HQT2.4 ionomer solution on bare and plasma-treated porous PTFE and PE substrates.

**Figure S2.** High-resolution O_1s_ spectra of (a) bare PTFE, (b) plasma-treated PTFE, (c) bare PE, and (d) plasma-treated PE substrates.

**Figure S3.** Schematic illustration of the plasma mode and reactive ion etching (RIE) mode in a Capacitively Coupled Plasma (CCP) system.

**Note:** As illustrated in **Figure S3**, two distinct operating modes can be achieved in a capacitively coupled plasma (CCP) system depending on the electrode configuration and sample position: plasma mode and reactive ion etching (RIE) mode. In this study, plasma mode was employed by placing the PE substrate on the grounded electrode while RF power was applied to the opposite electrode. Under these conditions, reactive oxygen species primarily reach the porous PE surface through diffusion, resulting in the introduction of oxygen-containing functional groups while minimizing ion bombardment, physical etching, and structural damage. In contrast, RIE mode places the sample on the powered electrode, where a self-DC bias is generated directly at the substrate surface, leading to strong directional ion bombardment and significant physical etching in addition to chemical modification. The corresponding operating parameters are summarized in **Table S1**.

**Figure S4.** Cross-sectional SEM images and average thickness variations of porous polyethylene (PE) supports under plasma mode and reactive ion etching (RIE) mode for 2 min.

**Figure S5.** Surface SEM images of (a-c) porous PE substrate and (d-f) PTFE substrate.

**Figure S6.** UV–vis transmittance spectra (300–800 nm) of (a) non-reinforced membrane (HQT2.4/Non) and RCMs with different porous substrate: (b) plasma-treated porous PE (HQT2.4/PE-P). (c) asymmetric plasma treated porous PE (HQT2.4/PE-P-H), (d) bare porous PE (HQT2.4/PE-N), (e) alcohol-mediated porous PTFE (HQT2.4/PTFE-A), (f) bare PTFE (HQT2.4/PTFE-N)

**Figure S7.** Low-magnification cross-sectional SEM image of the RCMs with different porous substrate. (a) bare porous PE (HQT2.4/PE-N) and (b) asymmetrically plasma-treated porous PE (HQT2.4/PE-P-H)

**Figure S8.** (a) XPS spectra obtained from the bare PE substrate, plasma-treated surface, and opposite (non-exposed) surface of the plasma-treated PE substrate. (b) Comparison of DI water contact angles for the bare PE substrate, plasma-treated surface, and opposite surface of the plasma-treated PE substrate. (c) EDS line-scan analysis showing changes in oxygen contents along the through-plane direction of the porous PE substrate.

**Figure S9.** Schematic illustrations of capillary-driven ionomer infiltration in porous PE substrates treated by (A) one-side plasma treatment and (B) dual-side plasma treatment. The SEM images shown on the right present magnified views of the PE substrate surface after formation of the first ionomer layer via capillary infiltration, prior to the second ionomer coating process.

**Figure S10.** Stress-strain curves of porous substrates and AEMs. (a) porous PE (9 µm), (b) porous PTFE (7 µm), (c) HQT2.4/Non (30 µm), (d) HQT2.4/Non (67 µm), (e) HQT2.4/PE-N (30 µm), (f) HQT2.4/PE-P (30 µm), (g) HQT2.4/PTFE-A (30 µm),

**Figure S11.** Representative photographs of tensile test specimens before and after mechanical testing. (a,d) Pristine porous PE substrate, (b,e) HQT2.4/PE-P, and (c,f) HQT2.4/PE-N. Images were taken before tensile testing (a–c) and after tensile failure (d–f).

**Figure S12.** Water uptake and dimensional change of RCMs with different temperatures. (a) HQT2.4/Non, (b) HQT2.4/PE-N, (c) HQT2.4/PE-P, and (d) HQT2.4/PTFE-A

**Figure S13.** Hydrogen crossover current densities of tested AEMs at 60 ℃ under fully-humidified condition. (a) HQT2.4-based AEMs, and (b) PiperION-A-based AEMs.

**Figure S14**. (a) Polarization curves measured under 1.0 M KOH at 60 °C using a multi-step chronopotentiometry for PE-reinforced membranes. Corresponding (b) high-frequency resistance (HFR) and (c) iR-free voltage extracted from electrochemical impedance spectroscopy measured at each current step.

**Figure S15.** Thickness-dependent extrapolation of the area-normalized HFR for the non-reinforced HQT2.4 membrane series to determine the non-membrane resistance contribution.

**Figure S16.** (a) Polarization curves measured under 1.0 M KOH at 60 °C using a multi-step chronopotentiometry for PiperION-A membranes. Corresponding (b) high-frequency resistance (HFR) and (c) iR-free voltage extracted from electrochemical impedance spectroscopy measured at each current step.

**Figure S17**. (a) Polarization curves of HQT2.4/PE-P-based AEMWE measured under 1.0 M KOH at 60 °C before and after the durability test. (b) Corresponding Nyquist EIS spectra measured at a current density of 1.0 A cm^-2^.

**Figure S18.** Digital Photograph of the HQT2.4/PE-P–based MEA after the durability test.

**Figure S19.** (a) Comparison of hydrogen crossover current densities for pristine HQT2.4/PE-P and HQT2.4/PE-P after the 1000 h durability test. (b) Cross-sectional SEM image of HQT2.4/PE-P after 1000 h durability operation.

**Figure S20.** TEM images of the Pt/C cathode catalyst (a) before and (b) after the durability test.

**Figure S21.** Chemical structure of HQT2.4

**Table S1.** Comparison of the two Capacitively Coupled Plasma (CCP) operating modes used in this study: plasma mode and reactive ion etching (RIE) mode.

| **Feature** | **Plasma Mode** | **Reactive Ion Etching (RIE) Mode** |
| --- | --- | --- |
| **Sample Placement** | Grounded electrode | Powered electrode |
| **Applied RF Power** | 25 W | 100 W |
| **Flow rate** | 10 sccm | 20 sccm |
| **Gauge Pressure** | 3.2 × 10^-1^ torr | 4.4 × 10^-1^ torr |
| **Frequency** | 50 kHz | 50 kHz |
| **Self-Bias Voltage**  **Ion Bombardment** | Negligible / None  Minimal to None | High / Strong negative bias  High-energy directional impacts |
| **Primary Mechanism** | Chemical functionalization dominant  (Radical diffusion) | Synergistic effect  (Physical impact + Chemical reaction) |
| **Etch Profile** | Isotropic | Anisotropic (Vertical trenches) |
| **Substrate Damage** | Minimal material loss | High damage potential |
| **Primary Purpose** | Enhances wettability via functionalization | Substantial material removal (Etching) |

**Note**: Plasma treatment was performed using a commercially available table-top plasma system (CUTE, Femto Science, Republic of Korea) based on a capacitively coupled plasma (CCP) configuration. The system is equipped with an RF generator operating in the frequency range of 20–100 kHz with a maximum power output of 100 W and can be operated over a pressure range from atmospheric pressure to 5 × 10⁻⁴ Torr. Process gases are supplied through a mass flow controller (MFC) with a maximum flow rate of 100 sccm. The plasma chamber dimensions are W 140 × D 200 × H 110 mm.
